# A Fatal Fate: A Medical Error Leading to Acute Methotrexate Toxicity

**DOI:** 10.7759/cureus.30659

**Published:** 2022-10-25

**Authors:** Sulhera Khan, Wajeeha Batool, Shabnam Naveed, Syed Masroor Ahmad

**Affiliations:** 1 Department of Internal Medicine, Jinnah Postgraduate Medical Centre, Karachi, PAK; 2 Internal Medicine, Jinnah Sindh Medical University, Karachi, PAK

**Keywords:** human error, high alert medications, folinic acid rescue, healthcare error, methotrexate poisoning

## Abstract

Methotrexate is an anti-metabolite, which is commonly utilized as an anti-cancer, anti-rheumatic, and anti-inflammatory drug used to treat a large variety of connective tissue disorders. Methotrexate when consumed at low doses for longer periods usually has a very limited side effect profile, however, accidental ingestion of large methotrexate doses is common which can result in a wide variety of adverse effects and can even result in fatal demise.

We, unfortunately, relate the incidence of a 75-year-old female, who unintentionally consumed 200mg of methotrexate instead of methylcobalamin because the pharmacist misunderstood the prescription. The patient presented to the Accident and Emergency (A&E) department of Jinnah Hospital, Karachi, with extensive hemorrhagic oral ulcers, maculopapular dermatitis, and inability to swallow, which further progressed to acute renal insufficiency, neutropenic sepsis, and respiratory distress. The patient was managed with leucovorin rescue therapy, intravenous rehydration, urinary alkalinization, neutropenic protocol, and oxygen support given her respiratory distress, and hemodialysis was arranged for her renal insufficiency. However, despite all these measures the patient met an unfortunate fate and expired.

Patients should be given accurate dosage directions, detailed textural information, and audio-visual resources. Additionally, symptoms of toxicity should be explained to all patients. Measures should be taken to minimize such unfortunate events in the future.

## Introduction

Methotrexate is a folate antagonist drug that works by inhibiting an enzyme Dihydro Folate Reductase (DHFR) that catalyzes the reduction of Dihydrofolate (DHF) to Tetrahydrofolate (THF) which is involved in the synthesis of DNA. It is widely used as an anti-metabolite, anti-neoplastic, immunosuppressant, and disease-modifying anti-rheumatic drug (DMARD) and has been effectively used in treating numerous conditions including malignancies, psoriasis, inflammatory bowel disease, rheumatoid arthritis and other connective tissue diseases [1].

Despite being therapeutically beneficial, it can produce serious and life-threatening side effects especially encountered with an accidental overdose. Side effects can range from nausea, vomiting, and mucocutaneous blisters to acute renal, pulmonary or hepatic failure [1]. Due to this reason, it has been added to the list of “high alert medicines” by The Institute for Safe Medication Practices (ISMP)-2020 [2]. Although side effects are usually encountered with chronic use, acute toxicity may result from the patient’s over-ingestion or a drug dispensing error [3]. Here we report a case of an unfortunate lady who consumed methotrexate accidentally due to the wrong delivery of medicine by the pharmacist.

## Case presentation

A 75-year-old female, a resident of Karachi, Pakistan, and a known case of hypertension for the last 25 years (untreated), presented to a local general physician for generalized weakness and fatigue. The patient was identified with anaemia on clinical history and examination. The dietary history revealed poor to no intake of meat and chicken due to financial reasons. She had been previously identified with vitamin B12 deficiency multiple times in the past and was treated accordingly. Owing to this and as a common healthcare practice by local physicians in society and non-compliance of the patients to get the investigations done due to financial restraints, the patient was prescribed intravenous methylcobalamin. The only medication regularly taken by the patient previously included non-steroidal anti-inflammatory agents (NSAIDs) due to occasional back and knee joint pain. The patient was advised to take injectable methylcobalamin intravenously on alternate days for 10 doses. The prescription was, unfortunately, wrongly comprehended by the pharmacist and she was given injectable methotrexate instead of methylcobalamin. The patient was administered intravenous methotrexate by untrained staff in a local clinic who did not verify the prescription before dispensing the drug and had no prior knowledge of pharmacokinetics, indications, or side effects of a high-alert medication like methotrexate. Consequently, the patient was administered four doses of methotrexate, 50mg each, on alternate days with total cumulative dose administration of 200mg.

Soon after the administration of the last dose of methotrexate, the patient’s general health and condition deteriorated with the development of oral stomatitis, anorexia, hair loss, and skin rash; her fatigue and malaise worsened. She presented to the emergency room with complaints of intractable, painful, and hemorrhagic oral ulcers, leading to difficulty and pain during chewing and swallowing resulting in anorexia. She also developed mild respiratory distress.

**Figure 1:**
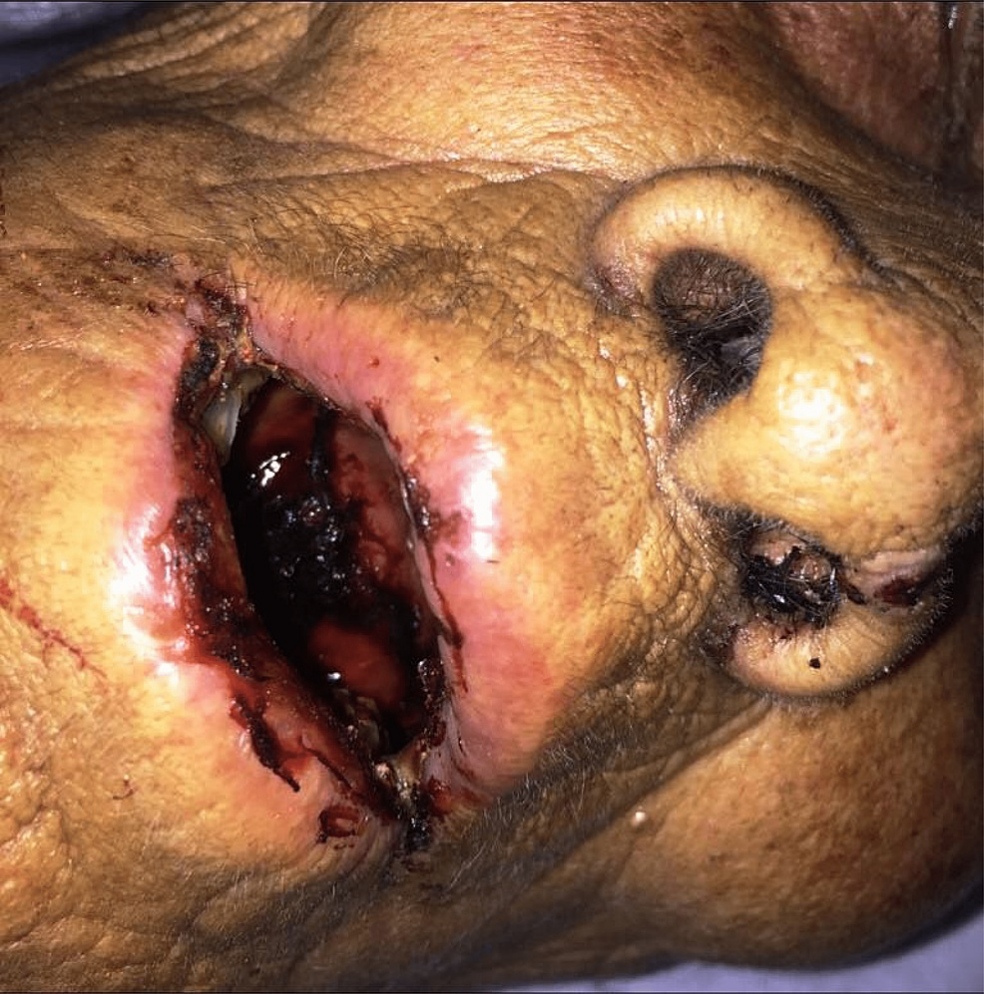
Patient showing extensive oral haemorrhagic ulcers

On examination, the patient appeared ill-looking and distressed although she was alert and well-oriented. The vitals recorded included a blood pressure of 110/70 mmHg, a pulse of 98 beats per minute, a respiratory rate of 25 breaths/min, and random blood sugar of 135 mg/dl. Oxygen saturation on room air was 84%. Her lips were painfully swollen, with crusting and bleeding from the ulcers (Figure 1).

**Figure 2:**
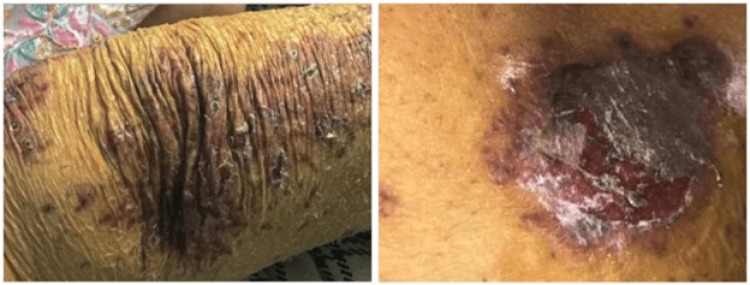
Vesiculopapular rashes developed after administration of methotrexate

She also had an eruption of vesicular-papular rash on her upper and lower extremities including her buttocks but sparing the palms and the soles; lesions were painful, non-pruritic, purple, and planar with irregular margins associated with drying of the surrounding skin (Figure 2). There were sub-centimetric lymph nodes in the submandibular region, however, no other lymph nodes were palpable. The neurological, abdominal, and cardiovascular examinations were not significant. The respiratory system, however, revealed tachypnea with harsh vesicular breathing. The laboratory investigations obtained on day one and the following days are listed in Table 1.

**Table 1:** Laboratory investigations obtained on days one, two, and three MCV: Mean corpuscular volume, TLC: Total leucocyte count, ANC: Absolute neutrophil count, ALT: Alanine aminotransferase, ALP: Alkaline phosphatase

Parameter	Day 1	Day 2	Day 3
Hemoglobin (g/dl)	11.8	11.9	10.2
MCV (fl)	78	80.5	78
Platelets (x10^9/L)	21	14	5
TLC (x10^9/L)	1.1	0.7	0.8
ANC (cells/uL)	660	420	440
Bilirubin (mg/dl)	2.26	3.6	2.6
ALT (U/L)	21	16	11
ALP (U/L)	67	55	39
Prothrombin time (seconds)	1.02	1.24	1.08
Creatinine (mg/dl)	4.89	5.54	6.86
Urea (mg/dl)	178	249	294
Sodium (mEq/L)	142	136	138
Potassium (mEq/L)	5.4	6.9	6.5
Chloride (mEq/L)	101	110	105
Bicarbonate (mEq/L)	12	8	8
Anion Gap	34.6	24.9	31.5

The patient was diagnosed with a case of acute methotrexate poisoning secondary to incorrect comprehension of the pharmacologic prescription. Methotrexate levels were planned to be checked but couldn’t be accomplished due to poor financial resources. Her management included immediate cessation of methotrexate, with the administration of injectable folinic acid 50mg daily as rescue therapy. For her respiratory distress, the patient was administered supplemental oxygen via face mask, with a target saturation of more than 94%. To enhance renal excretion of methotrexate; intravenous hydration with crystalloids was initiated, along with urinary alkalization with 50mEq of sodium bicarbonate per litre of fluid. Urine output was strictly monitored to ensure an output of at least 100ml/hr. Mouthwash containing folinic acid and a topical coating agent before each meal were advised for mouth ulcers. Daily dressing with ointments containing antibiotics and steroids for cutaneous rash was performed. Given her neutropenia, prophylactic measures were taken; the patient was isolated, and a contact precautions protocol was initiated. Blood and urine cultures were sent, and the patient was started on broad-spectrum non-nephrotoxic antibiotics prophylactically. Measures were taken to make Granulocyte colony-stimulating factor (G-CSF) readily available but couldn’t be achieved due to a lack of resources. Due to a severely reduced platelet count, the patient was transfused with random donor manual platelets prophylactically to prevent her from life-threatening bleeding.

During her course of admission, the general condition of the patient deteriorated with the worsening of her mucositis and dermatitis. Her renal status did not improve with hydration and alkalization and the patient underwent one session of hemodialysis which regrettably was not successful in preventing the patient from a fatal fate and demise on the third day of her admission.

## Discussion

Methotrexate was traditionally used as an anti-cancer agent but now is widely available as an immunotherapy drug used in a wide variety of autoimmune and inflammatory disorders [1]. In many connective tissue diseases, such as rheumatoid arthritis, methotrexate is used for a longer period than other drugs [4]. It has been seen that at low doses (7.5-10 mg), such as those used in rheumatoid arthritis, the side effects of methotrexate are controlled, with minimal derangements of liver function tests and enzymes [4]. It is also observed that with the supplementation of methotrexate antidote; folic acid, patients are seen to experience significantly reduced adverse effects [4].

On the other end of our discussion, our patient was identified with anaemia at a local clinic on clinical history and examination. She was prescribed methylcobalamin. However, due to incorrect comprehension of the prescription and erroneous administration of the drug by the administrator, the patient was given a high (200mg) dose of methotrexate. Another study conducted by Yazici et al. reporting a population of 248 patients taking methotrexate for rheumatoid arthritis showed most patients were free of side effects upon consumption of the drug for prolonged periods at a smaller less toxic dose [4]. Therefore, it is significant to carefully monitor for toxicity and side effects of methotrexate especially if consumed at very high toxic doses which can result in a fatal fate [5]. Many adverse effects have been associated with methotrexate overdose that includes headache, anorexia, stomatitis, dermatitis, alopecia, pancytopenia secondary to bone marrow suppression, derangements of liver function tests which may progress to hepatic fibrosis, pneumonitis, acute tubular necrosis, and even death [5]. In a similar case study by Bidaki et al. where the patient, a 68-year-old woman accidentally ingested one tablet of methotrexate instead of digoxin every day [6]. The patient had bi-cytopenia (anaemia and thrombocytopenia) with a normal leukocyte count, mucocutaneous lesions, and hemorrhagic oral ulcers [6]. The patient was treated with leucovorin (folinic acid) rescue therapy which resulted in an improvement in the patient’s presenting complaints after 10 days [6]. In the same study, the author also discusses another medical error where the prescription was incorrectly comprehended by the pharmacist leading to the erroneous consumption of 1.25mg of methotrexate every day by an 88-year-old woman for one month in place of digoxin [6]. The patient presented to the emergency department with nausea, vomiting, diffuse erythematous rash involving the back, oral ulcers, pancytopenia, dyspnea along with deranged renal function tests. Despite resuscitation with folinic acid, antibiotics, and granulocyte stimulating factor, the patient embraced a fatal course due to her age and underlying cardiac status; a similar end was seen in our patient [6].

It is seen that among all features of methotrexate toxicity, the development of mucocutaneous and oral ulcers determines systemic toxicity [6]. Methotrexate can cause renal failure due to its direct toxic effects on the renal tubules or due to acute tubular necrosis [7]. The renal failure associated with methotrexate toxicity is further exacerbated by the reduced clearance of the drug [7]. Methotrexate-induced nephrotoxicity is managed with hydration, urinary alkalization, leucovorin, monitoring of serum creatinine and methotrexate levels, and hemodialysis [7]. It is, therefore, cardinal to identify toxicity and start management as early as possible to prevent the demise of the patients [8]. Among factors exacerbating the risks of toxicity, the presence of renal disease and concomitant consumption of drugs interacting with metabolism and excretion of methotrexate tops the list. The commonly used drugs interacting with methotrexate include non-steroidal anti-inflammatory agents (NSAIDs), aspirin, sulfonylureas, and penicillin which displace methotrexate from plasma proteins and increase its free serum levels. Similarly, drugs like trimethoprim, triamterene, and pyrimethamine also result in toxicity when combined with methotrexate because of their similar mechanism of action [8]. Our patient was consuming NSAIDs which may have contributed to exacerbating methotrexate toxicity. For adequate management and reversal of methotrexate toxicity, it is important to halt methotrexate and administer a leucovorin rescue dose (10mg/m^2^ every six hours and increased to 100 mg/m^2^ if methotrexate levels fail to fall or there is a development of methotrexate-induced nephrotoxicity) which should be continued until the levels of methotrexate fall below 0.05 micromolar with intravenous fluid replenishment, urinary alkalization with sodium bicarbonate, and granulocyte-colony stimulating factors (G-CSF) [8]. To prevent medical errors, it is important for the clinician when initiating methotrexate to provide an elaborate and clear prescription to the patient mention the number of tablets to be consumed, also using the same strength of the tablets, the timing of administration, and fix a day for methotrexate and a day for folic acid administration. It is usually remembered as M for Methotrexate and M for Monday, F for Folic acid and F for Friday [9]. It is also imperative for the pharmacist to properly read the prescription and discuss it with the clinician if required to ensure proper dosing, possible interactions, and review of adverse events. Dosing and administration fall under the department of nursing, it is important that a trained staff comprehends the prescription clearly and confirms from the healthcare professional before administering the dose. The staff should be well-informed regarding the potential adverse events and should keep the clinicians updated in case any adverse events arise for correction. Interprofessional communication is crucial in terms of preventing accidental toxicities related to methotrexate. It is also important to hold regular meetings with the patients for follow-ups and, inquiring any development of side effects. Patients should be explained the side effect profile before administration of the drug, along with, the provision of proper education material in the form of either textural or audio/visual documents [5]. It is also important to perform regular laboratory investigations, including full blood count to look for bone marrow suppression, liver function tests, and renal function tests. These investigations should be performed at baseline before the commencement of the drug, at two to four weeks, and then monthly, and finally to three monthly [5]. Radiological investigations, such as a chest X-ray should also be recorded at baseline [5]. The patients should be investigated for polypharmacy and the list of drugs consumed by the patients should be provided to the physician [5]. Methotrexate is teratogenic with a wide range of fetotoxic effects; therefore, all women of childbearing age must be counselled regarding contraception and a dual contraceptive method should be used. Mothers should also be counselled to avoid breastfeeding [5].

Methotrexate is a highly effective and commonly used drug for cancers and autoimmune disorders in society. With its pharmacologic effects on several diseases, it is also important to watch out for side effects and monitor patients for any accidental overdose and toxicity. For individuals using long-term methotrexate, it is crucial to concomitantly administer folic acid to prevent pancytopenia, neutropenic sepsis, mucocutaneous lesions, and renal and hepatic toxicity.

## Conclusions

Our case report highlights an important domain of the healthcare system that requires understanding and safe practice of prescribing medications, especially those with a large range of adverse effects. Methotrexate is one of those drugs that can be fatal when consumed at higher doses, therefore it is important to be vigilant and maintain regular follow-ups of patients on methotrexate to decrease the incidence of toxicity.

## References

[1] Bedoui Y, Guillot X, Sélambarom J (2019). Methotrexate an old drug with new tricks. Int J Mol Sci.

[2] (2022). Methotrexate FDA Institute for Safe Medication Practices (ISMP). https://www.ismp.org/recommendations.

[3] Madke B, Singh A (2015). Acute methotrexate toxicity. Indian J Drugs Dermatol.

[4] Yazici Y, Sokka T, Kautiainen H, Swearingen C, Kulman I, Pincus T (2005). Long term safety of methotrexate in routine clinical care: discontinuation is unusual and rarely the result of laboratory abnormalities. Ann Rheum Dis.

[5] (2014). Methotrexate - safe prescribing - once a week. http://www.saferx.co.nz/assets/Documents/full/06640c86a2/methotrexate.pdf.

[6] Bidaki R, Kian M, Owliaey H, Babaei Zarch M, Feysal M (2017). Accidental chronic poisoning with methotrexate; report of two cases. Emerg (Tehran).

[7] Al-Turkmani MR, Law T, Narla A, Kellogg MD (2010). Difficulty measuring methotrexate in a patient with high-dose methotrexate-induced nephrotoxicity. Clin Chem.

[8] Tan KW, Tay YK (2011). A case of acute methotrexate toxicity. Ann Acad Med Singap.

[9] Amissah-Arthur MB, Baah W (2020). Methotrexate-induced pancytopenia and mucositis caused by medication error. Ghana Med J.

